# Renal Cell Carcinoma Diagnosis After Initial Detection on Screening Mammogram

**DOI:** 10.7759/cureus.10428

**Published:** 2020-09-13

**Authors:** Quan D Nguyen, Hyunjoo Ko, Angelica S Robinson, Anne E Lee, Jing He

**Affiliations:** 1 Radiology, University of Texas Medical Branch, Galveston, USA; 2 Pathology, University of Texas Medical Branch, Galveston, USA

**Keywords:** renal cell metastasis, clear cell cancer, carcinomas renal cell, breast mass, renal cancer, breast cancer, breast metastasis, renal carcinoma recurrence, post-nephrectomy renal cell carcinoma, distant metastasis

## Abstract

Renal cell carcinoma (RCC) defines a varied class of primary renal neoplasms which arise from the renal cortex. Because RCC often progresses silently to a very advanced metastatic stage, the majority of RCC cases are diagnosed either incidentally on abdominal imaging or upon presentation of invasive disease at metastatic sites. This case profiles a 57-year-old woman with distant history of resected RCC who presented with a posterior breast mass that was diagnosed as metastatic recurrence of RCC through mammogram, ultrasound, and core biopsy. Although the breast is an unusual site for metastasis, clinicians should consider metastatic RCC as a possible etiology when evaluating women with history of RCC and a newly discovered breast mass.

## Introduction

Renal cell carcinoma (RCC) encompasses a varied class of primary renal neoplasms, which arise from the renal cortex and include clear cell carcinoma, papillary carcinomas, and other subtypes. RCC has been associated with a wide array of aberrant gene alterations, including loss of function at several points along chromosome 3p and gain of function at chromosome 5q [1]. Although RCC can manifest as one of the features of Von Hippel-Lindau syndrome, the majority of RCC cases arise sporadically. Significant risk factors for RCC include but are not limited to smoking, hypertension, obesity, and exposure to phenacetin and some toxic industrial byproducts, such as cadmium [2,3].

The majority of RCC cases are diagnosed either incidentally on abdominal imaging or upon presentation of invasive disease at common metastatic sites, such as the lung, bone, and liver. The traditional RCC triad of hematuria, flank pain, and palpable abdominal mass is uncommon. For renal masses, both ultrasound and abdominal CT are used to determine the likelihood of malignancy; however, CT remains the gold standard for diagnosis. For RCC discovered both in the kidney and at distant metastatic sites, core biopsy and pathological examination are performed to confirm diagnosis. Significant morphological diversity exists among RCC subtypes, and tissue composition within a single RCC tumor is often heterogeneous. For example, cells with large quantities of clear cytoplasm, tubular growth, spindle cells, and thick sheets of cells have all been described in pathology samples of RCC [4]. In cases of RCC with indeterminate or mixed histological features, immunohistochemical staining may be utilized to clarify the final diagnosis. Staging of all variants of RCC uses CT imaging findings to classify tumors by the tumor, node, and metastasis staging system.

Surgical resection of early-stage localized RCC is considered curative with appropriate postoperative surveillance measures and judicious follow-up. However, most patients with RCC remain asymptomatic until symptoms of advanced metastatic spread develop, so they often require systemic treatment at the time of diagnosis. Distinguishing clear cell RCC from non-clear cell RCC guides treatment selection. For clear cell RCC, the standard immunotherapy regimen is usually a combination of programmed cell death 1 protein (PD-1) checkpoint inhibitors, cytotoxic T-lymphocyte antigen 4 (CTLA-4) checkpoint inhibitors, and vascular endothelial growth factor (VEGF) inhibitors [5]. For non-clear cell RCC, treatment is tailored to the specific RCC subtype and may include immunotherapy, antiangiogenic therapy, or platinum-based chemotherapy.

## Case presentation

A 57-year-old post-menopausal woman presented to the clinic for a well-woman examination. Her past medical history includes hypertension, hypothyroidism, benign breast cyst, leiomyoma, and RCC in the left kidney capsule. Surgical history includes breast biopsy for benign breast cyst, hysterectomy and bilateral salpingo-oophorectomy for leiomyoma, and left nephrectomy for RCC. The patient has family history of breast cancer in her mother, but no smoking history.

**Figure 1:**
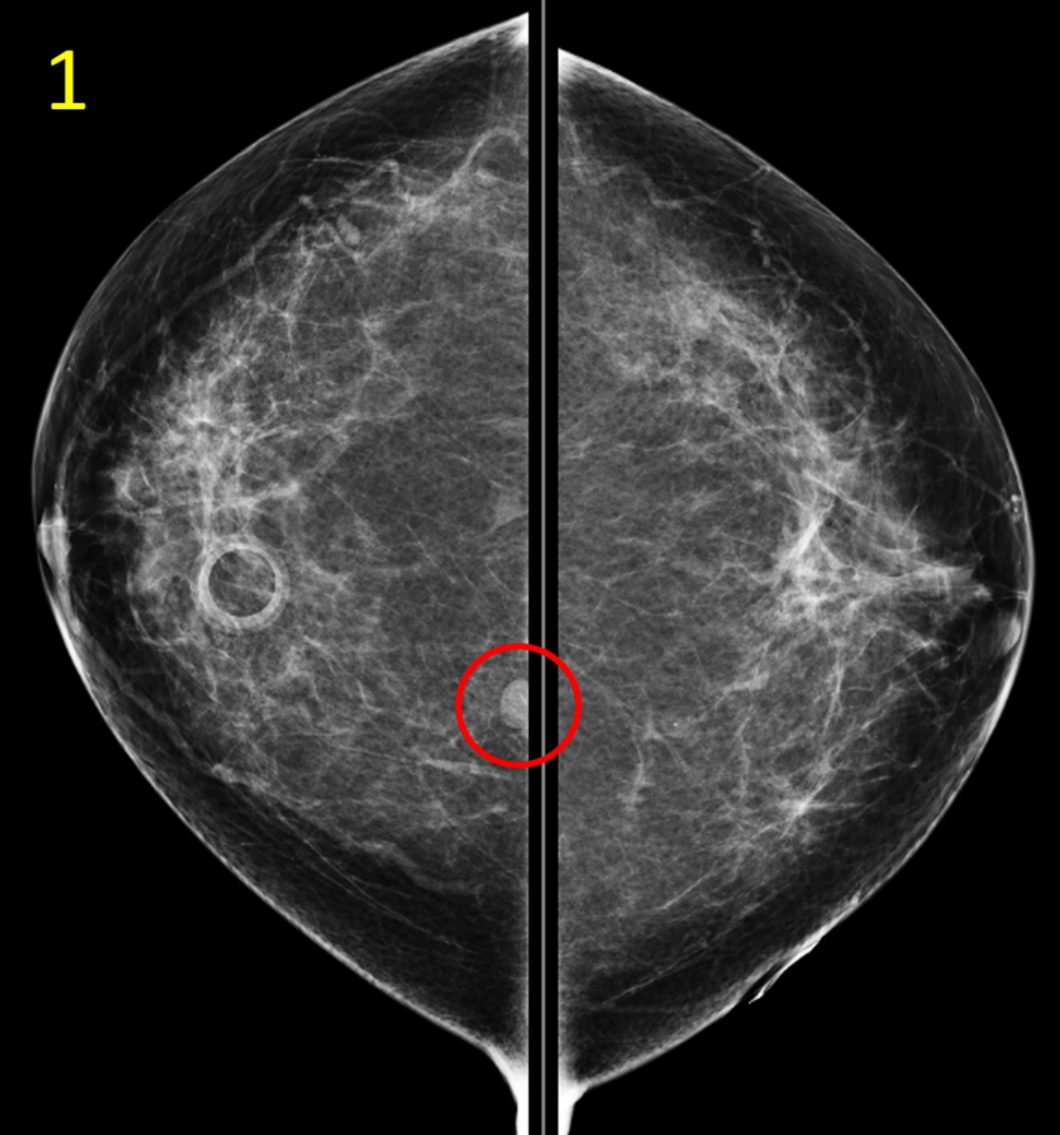
Initial Screening Mammogram of the Bilateral Breasts, Craniocaudal (CC) View This CC view of the initial screening mammogram depicts a subcentimeter mass (red circle) in the posterior upper inner quadrant of the right breast at 1 o’clock, 14 cm from the nipple.

**Figure 2:**
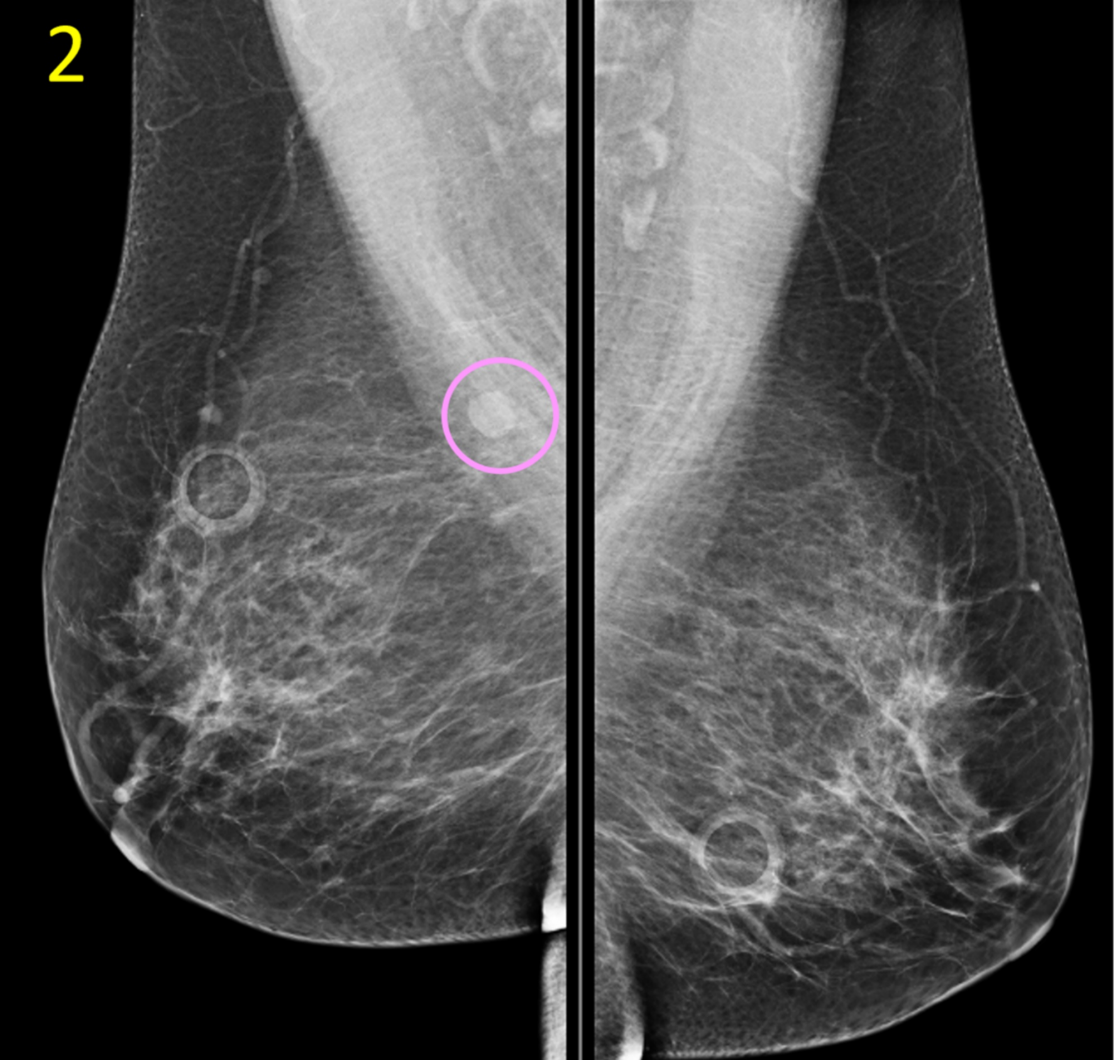
Initial Screening Mammogram of Bilateral Breasts, Mediolateral Oblique (MLO) View This MLO view of the initial screening mammogram demonstrates the posterior placement of the subcentimeter breast mass (pink circle) in the upper inner quadrant at 1 o’clock, 14 cm from the nipple.

Routine physical exam was unremarkable, with no palpable masses in either breast bilaterally. The patient was scheduled for a routine screening mammogram, which revealed a concerning mass of approximately 10 mm diameter in the upper inner quadrant of the breast (Figures 1, 2).

**Figure 3:**
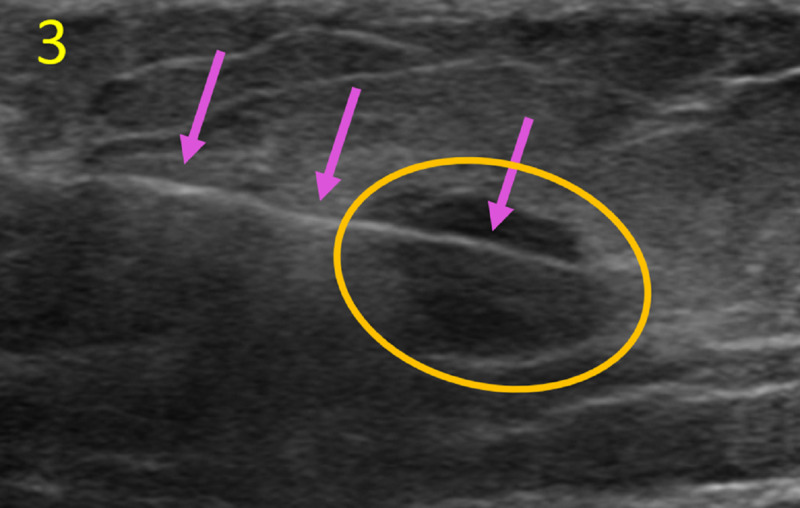
Ultrasound-Guided Biopsy of Right Breast Mass This ultrasound depicts the biopsy needle (pink arrows) sampling the oval right breast mass (orange circle) discovered on screening mammogram. The mass was oriented parallel to the skin with circumscribed margins.

Subsequent imaging revealed a solid, oval mass (9 mm x 8 mm x 6 mm) with circumscribed margins, categorized as Breast Imaging-Reporting and Data System Category 4B (Figure 3).

**Figure 4:**
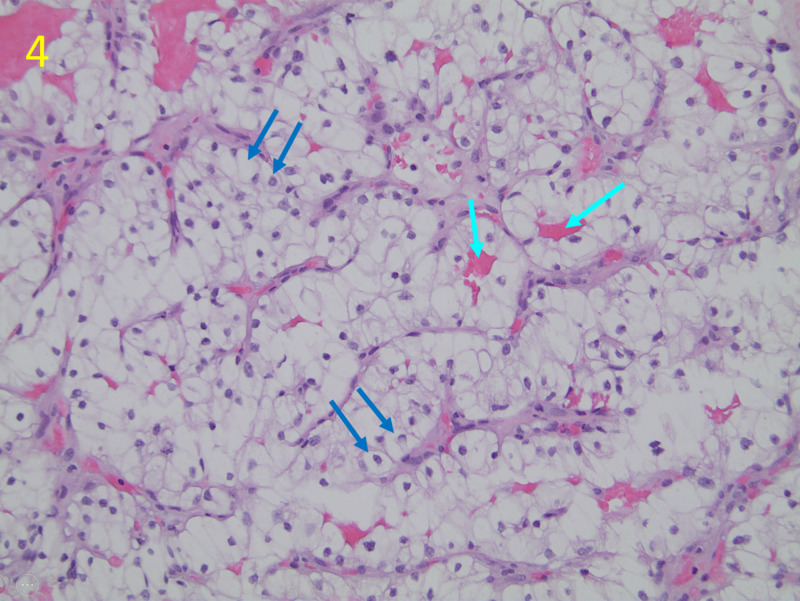
Hematoxylin and Eosin (H&E) Stain of Biopsy Tissue From Right Breast Mass This H&E stain of the tissue biopsy site demonstrates classic histological findings of clear renal cell carcinoma, including bountiful clear cytoplasm with distinct cell boundaries (dark blue arrows) and networks of thin-walled, “chicken-wire” vasculature (cyan arrows). Magnification is ×200.

**Figure 5:**
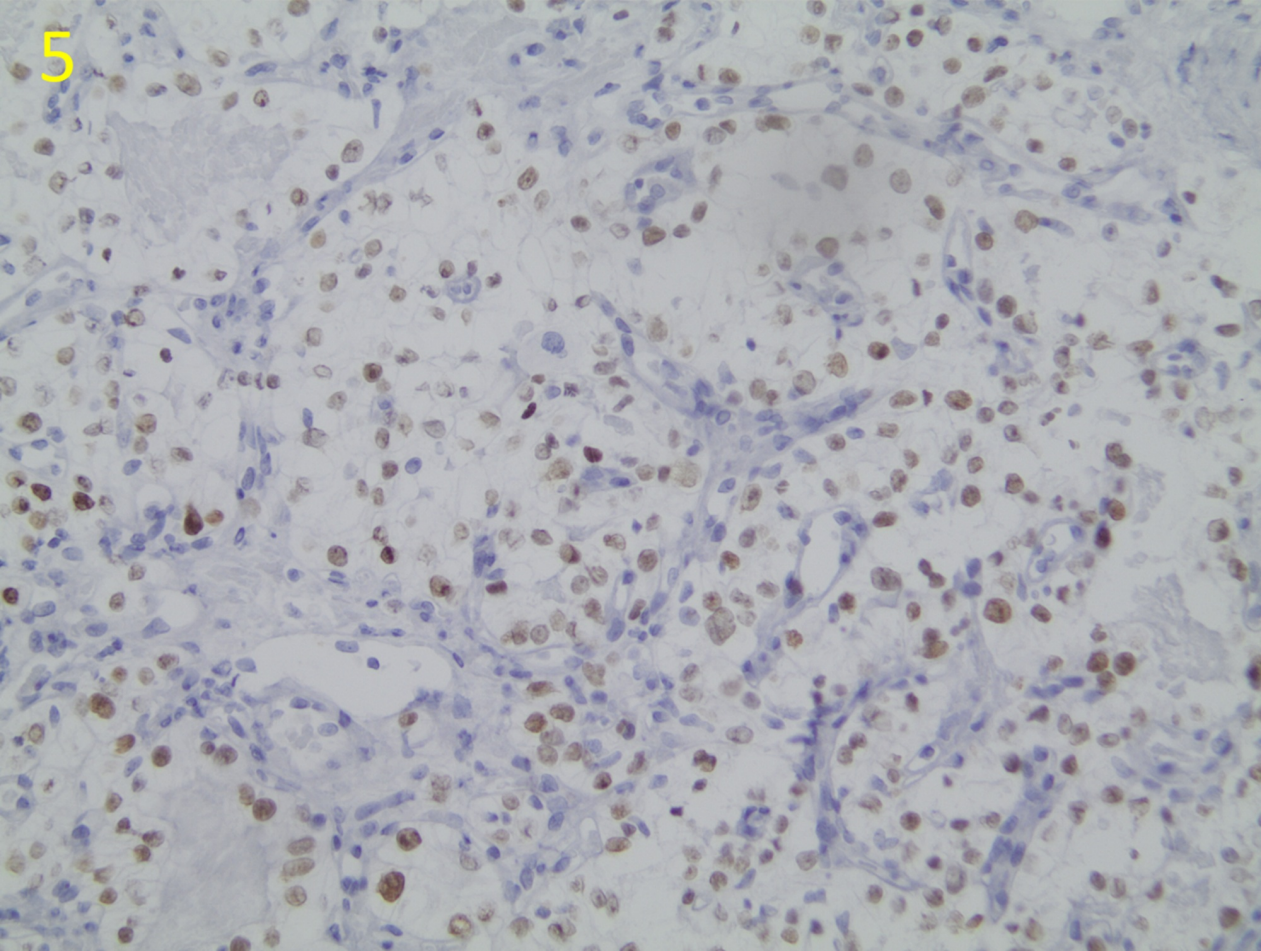
Targeted Immunohistochemical Stain of Paired-Box 8 (PAX8) Biomarker This immunohistochemical stain of the biopsied tissues at ×200 magnification demonstrates positive staining of PAX8 nuclear stain, which is a biomarker used to identify certain epithelial and neuroendocrine tumors such as renal cell carcinoma. Brown staining in the tissue indicates presence of the selected cell marker.

Cytology and immunohistochemical staining of biopsy specimens identified metastatic renal clear cell carcinoma (Figures 4, 5).

**Figure 6:**
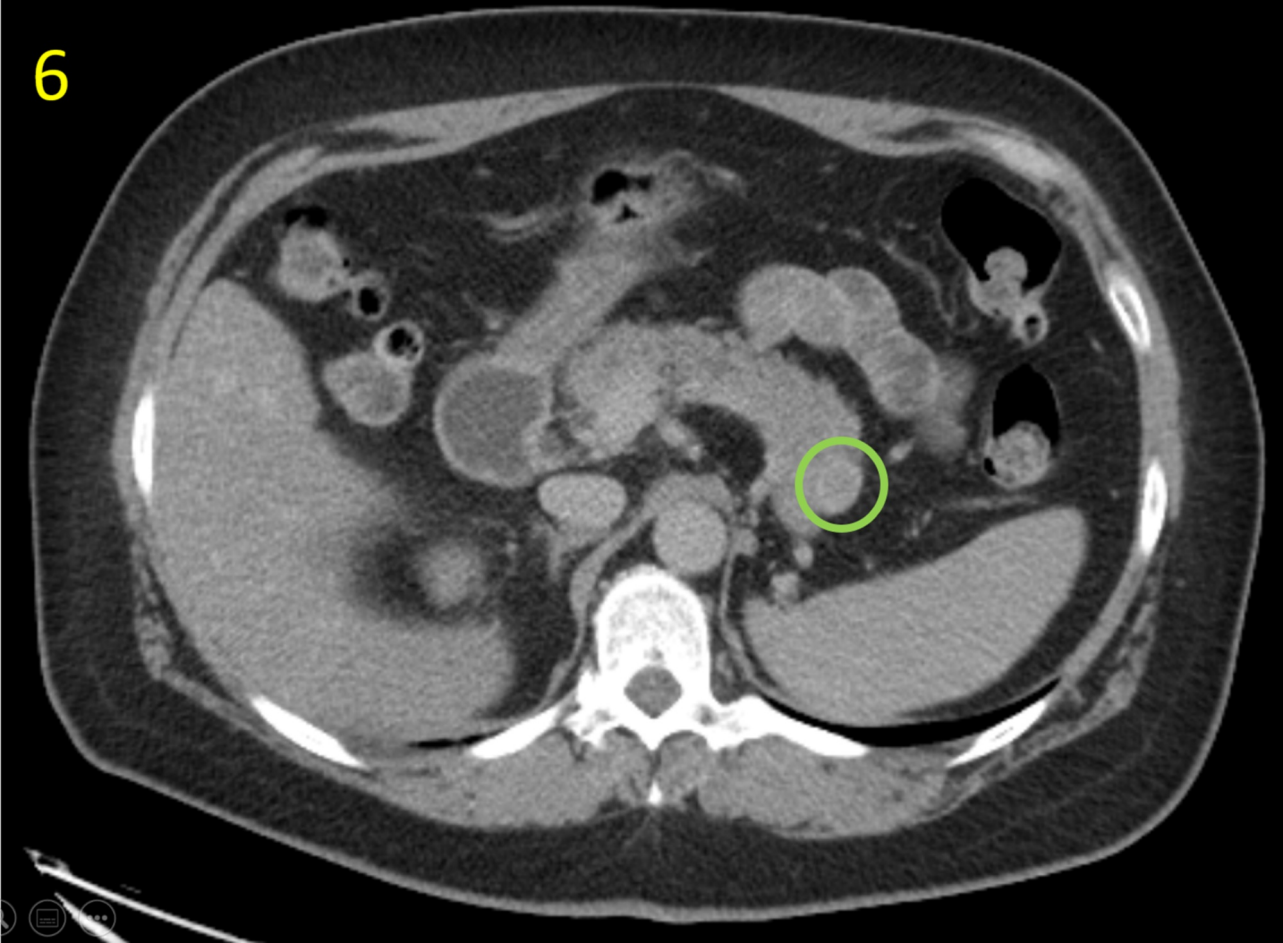
Non-Contrast Abdominal CT of Pancreas This abdominal CT demonstrates a mass suspicious for metastasis in the tail of the pancreas (green circle). The study was limited due to lack of intravenous contrast.

**Figure 7:**
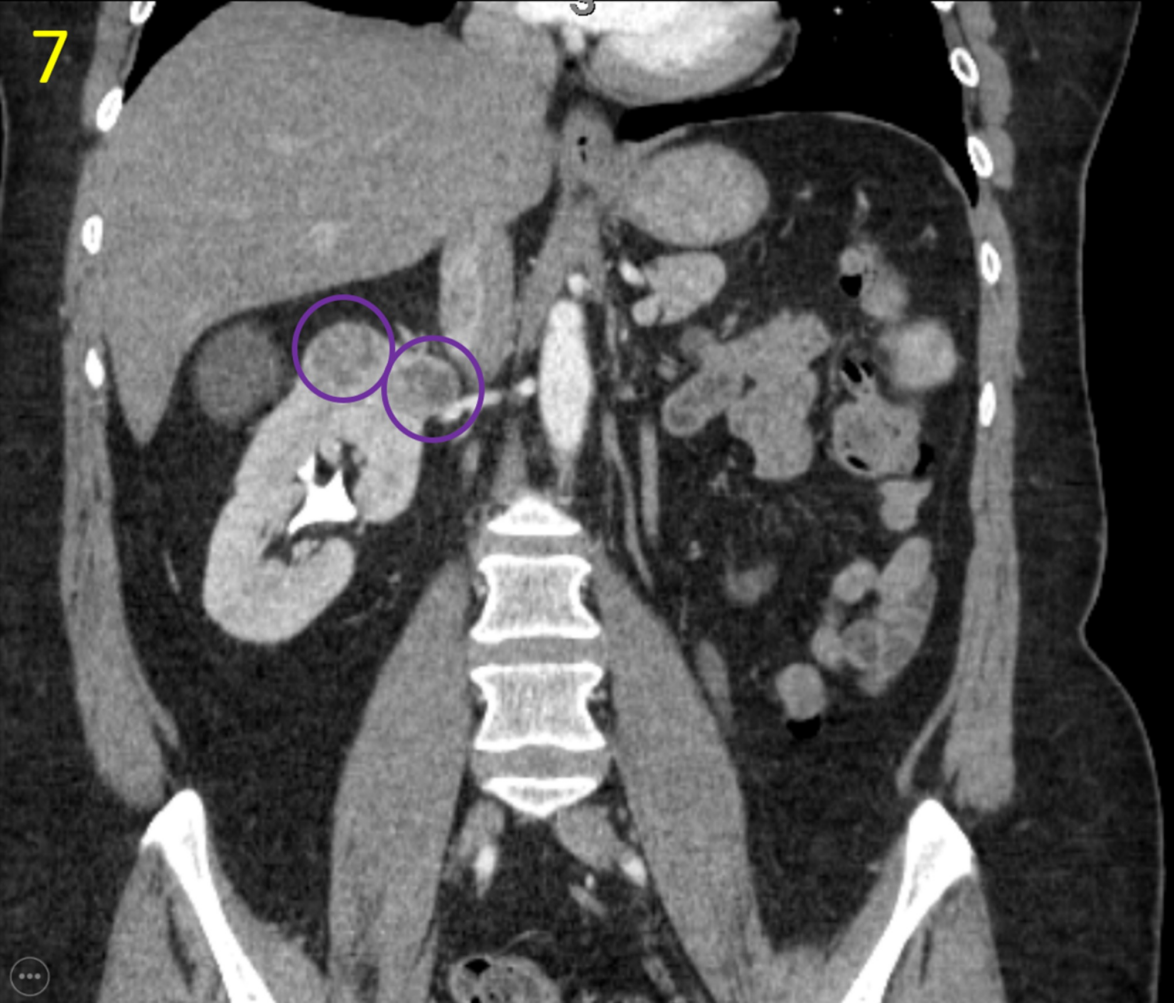
Coronal Abdominal CT This coronal abdominal CT demonstrates several masses in the right kidney suspicious for metastasis (purple circles). There was no invasion of the right renal vein nor any signs of recurrence in the left kidney bed where the radical nephrectomy had been performed.

Following histology and cytology assessment, non-contrast abdomen and thorax CT scans were ordered to evaluate for possible metastases. Multiple solid lesions suspicious for malignancy were noted in the tail of the pancreas and remaining right kidney (Figures 6, 7).

After diagnosis, the patient was started on sunitinib, a multitargeted tyrosine kinase inhibitor with antitumor and antiangiogenic activities. She has completed five cycles of immunotherapy. Over the course of her treatment, she developed hypothyroidism and symptoms of severe gastrointestinal and skin toxicity, which were controlled after immunotherapy dose adjustment and hormone supplementation. Excluding one right adrenal mass that was found to have increased in size on the most recent study, sequential CT imaging found decreased size and cystic transformation of suspected metastatic lesions. She currently continues to receive sunitinib, and she is followed by medical oncology and endocrinology.

## Discussion

RCC is a rare epithelial cancer that often progresses silently to a very advanced metastatic stage upon initial clinical presentation. Some patients who were thought to have been successfully treated for RCC experience recurrence years or decades after their initial diagnosis and treatment. The most common sites of metastasis are lung, bone, and liver; however, cases of late recurrence RCC have been diagnosed in unusual distant metastatic sites, including the skin, heart, and parotid glands [6]. The breast is a rare site for metastasis from any type of malignancy, and an initial diagnosis of RCC discovered within the breast is especially unusual [7-9].

When discovered as metastases distant from the kidney, RCC is difficult to identify on imaging. Malignancies that have metastasized to the breast, including RCC, most often appear as circumscribed round or oval-shaped masses, which appear hypoechoic on ultrasound and hyperdense on CT [10]. Spiculation and calcification are uncommon in metastatic breast masses as compared to primary breast malignancy [11]. Although these patterns on imaging may raise suspicion for breast metastasis, comprehensive literature review shows that these findings are not reliable enough to distinguish RCC or other metastatic disease from primary breast malignancy or benign breast conditions [10,12]. Biopsy with cytology and immunohistochemical staining is necessary to identify the malignancy and guide treatment planning. Although significant morphological diversity exists among subtypes of RCC, the most common RCC is clear cell carcinoma, which characteristically appears as nests or sheets of well-defined cells with large amounts of clear cytoplasm and prolific arborizing vasculature.

As demonstrated in this case, accurate diagnosis of a breast mass is crucial to designing an effective therapeutic approach. Treatment for primary breast malignancy typically includes surgical resection with adjuvant radiation or endocrine therapy. In contrast, metastatic RCC is managed primarily through systemic antiangiogenics and targeted immunotherapy; surgical intervention is usually limited to cytoreductive nephrectomy in select patients [13]. Understanding the etiology of a malignant breast mass also strongly influences the expected outcomes for the patient. Prognosis of breast cancer varies widely based on stage of disease and treatment responsiveness; patients with localized disease may have excellent, long-lasting outcomes. Conversely, metastatic RCC has an expected prognosis of approximately 28 months [14]. However, patients who were previously treated for limited-stage with a subsequent later recurrence of distant metastatic disease tend to have better outcomes than those patients whose initial diagnosis is metastatic RCC [15].

## Conclusions

Patients with distant history of treated RCC may experience late recurrence of disease at unusual sites, including the breast. In the case of this patient, radiology and pathology collaboration diagnosed a suspicious breast mass and established a final diagnosis of clear cell RCC. Clinicians should consider metastatic RCC as a possible etiology when evaluating women with history of RCC and a newly discovered breast mass.
